# Identifying microRNA targets in different gene regions

**DOI:** 10.1186/1471-2105-15-S7-S4

**Published:** 2014-05-28

**Authors:** Wenlong Xu, Anthony San Lucas, Zixing Wang, Yin Liu

**Affiliations:** 1 Department of Neurobiology and Anatomy, University of Texas Health Science Center at Houston, Fannin Street, Houston, USA; 2 Department of Epidemiology, University of Texas MD Anderson Cancer Center, Holcombe Boulevard, Houston, USA; 3 University of Texas Graduate School of Biomedical Sciences, Bertner Avenue, Houston, USA

## Abstract

**Background:**

Currently available microRNA (miRNA) target prediction algorithms require the presence of a conserved seed match to the 5' end of the miRNA and limit the target sites to the 3' untranslated regions of mRNAs. However, it has been noted that these requirements may be too stringent, leading to a substantial number of missing targets.

**Results:**

We have developed TargetS, a novel computational approach for predicting miRNA targets with the target sites located along entire gene sequences, which permits finding additional targets that are not located in the 3' un-translated regions. Our model is based on both canonical seed matching and non-canonical seed pairing, which discovers targets that allow one bit GU wobble. It does not rely on evolutionary conservation, so it allows the detection of species-specific miRNA-mRNA interactions and makes it suitable for analyzing un-conserved gene sequences. To test the performance of our approach, we have imported the widely used benchmark dataset revealing fold-changes in protein production corresponding to each of the five selected microRNAs. Compared to well-known miRNA target prediction tools, including TargetScanS, PicTar and MicroT_CDS, our method yields the highest sensitivity, while achieving a comparable level of accuracy. Human miRNA target predictions using our computational approach are available online at http://liubioinfolab.org/targetS/mirna.html

**Conclusions:**

A simple but powerful computational miRNA target prediction method is developed that is solely based on canonical and non-canonical seed matches without requiring evolutionary conservation of the target sites. Our method also expands the target search space to different gene regions, rather than limiting to 3'UTR only. This improves the sensitivity of miRNA target identification, while achieving a comparable accuracy with existing methods.

## Background

MicroRNAs (miRNAs) are endogenous approximately 22 nucleotide RNA molecules that play important gene-regulatory roles in plants and animals [1]. These small RNA molecules exert their regulatory effects on target gene mRNAs by inhibiting protein translation and/or promoting mRNA degradation. They are one of the most abundant classes of gene-regulatory molecules in mammals [2], with more than two thousand distinct miRNAs having been confidently identified in human [3]. It has been estimated that at least 30% and perhaps as many as 60% of mRNAs are subject to post-transcription miRNA-mediated regulation [4]. It has also been shown that a single miRNA can modulate the expression levels of several hundred to thousands of different mRNAs [5]. Therefore, to fully understand the roles miRNA play in regulating different biological processes, one essential step is to determine which mRNAs are targeted for miRNA regulation.

In the past decade, dozens of miRNA target prediction programs for mammalian genomes have been developed, including TargetScanS [4,6-8], PicTar [9], MicroT_CDS [10,11], miRanda [12,13], RNAhybrid [14], MirTarget2 [15], TargetMiner [16] and others [17-21]. The majority of these algorithms are based on the assumption that miRNAs target recognition requires conserved Waston-Crick pairing to the 5' region of the miRNA centered on nucleotides 2-7, which is known as the miRNA "seed". This notion has resulted in a biased focus on special types of seed-matched sites within the 3' untranslated regions (3'UTRs) of targeted mRNAs [22,23]. However, many experimental results show that some "non-seed" miRNA target sites are highly biologically functional [24-26]. These non-seed sites contain single mismatches, GU wobbles, insertions or deletions in the seed-match regions. Besides the seed match "rule", most of the existing computational methods rely on evolutionarily conservation of putative target sites for target identification. However, there is no evidence showing that target sites must be evolutionarily conserved [24]. Identification of mRNAs and proteins that are upregulated upon inhibition or the removal of an endogenous miRNA demonstrate that non-conserved targeting is even more widespread than conserved targeting [5,27]. In addition, we note that most investigations into metazoan miRNA regulation have been focusing on searching for target sites in 3'UTRs. However, experiments have shown that targeting can occur in the 5' untranslated regions (5' UTRs) and the open reading frame (ORF) as well [28]. Recently, Hafner et al. found that of the exonic target regions, 50% of target sites correspond to coding sequences (CDSs), compared with only 46% to 3'UTRs [29]. Chi et al. also applied a high-throughput approach for isolating Argonaute-bound target sites, indicating that target sites in CDSs are as numerous as those located in 3'UTRs [30].

In this article, we introduce a simple but powerful miRNA target prediction method that is solely based on canonical seed pairing and non-canonical seed matches. Our method does not require stringent seed pairing or evolutionary conservation in searching for human miRNA target sites. In addition, we perform our search on the entire gene sequence (including promoters, 5'UTRs, CDSs, and 3'UTRs) rather than limiting the search space to the 3'UTRs only. We assessed our method based on a set of miRNA targets identified by the pSILAC method [5]. It is found that, without applying complicated scoring schemes and considering evolutionary conservation of the target sites, our method successfully yielded the largest number of true targets while achieving a comparable level of accuracy, among all the methods we compared.

## Results and discussion

### Comparison of signal-to-noise ratios

**Figure 1:**
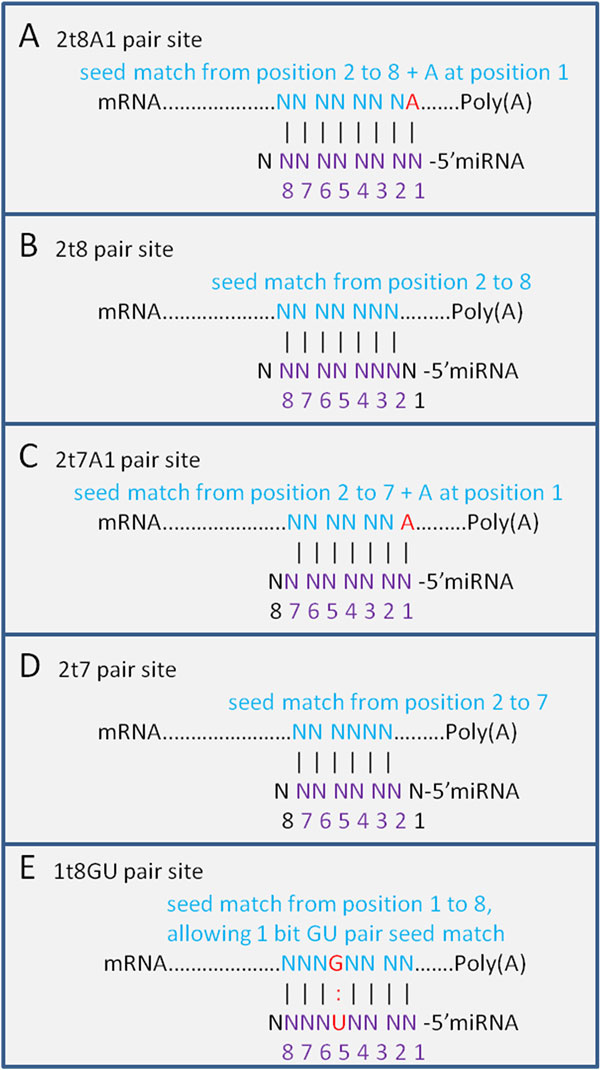
**Types of seed matches**. Five different types of seed matches used in our study, including canonical seed match types 2t8A1, 2t8, 2t7A1 and 2t7, and a non-canonical 1t8GU wobble type.

**Figure 2:**
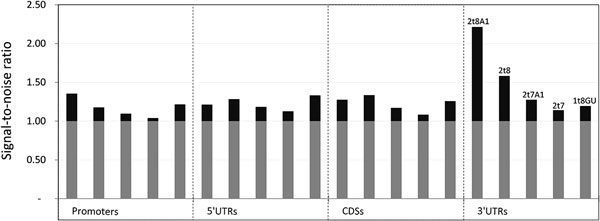
**Signal-to-noise ratio**. Signal-to-noise ratio for five different types of seed matches in four different gene regions.

The five types of seed matches used in our study are illustrated in Figure 1. We used the miRWalk dataset to calculate the signal-to-noise ratios for each type of seed match located in different gene regions (see Methods section for details). Figure 2 compares the signal-to-noise ratios of the five types of seed matches in different gene regions. The weights for each seed match type were then calculated and listed in Table 1. It can be seen that seed matches located in 3'UTRs have the highest signal-to-noise ratios, followed by those in CDSs and 5'UTRs, while the seed matches in the Promoter regions have the lowest values. We can see that the signal-to-noise ratio of type 2t8A1 in 3'UTRs is the highest, with 2t8A1 > 2t8 > 2t7A1 > 2t7 in 3'UTRs, which is consistent with previous conclusions [23]. The results also show that the signal-to-noise ratio of type 1t8GU is even higher than 2t7 in 3'UTRs, indicating that the 1t8GU seed matches in 3'UTRs may represent important biologically functional sites. This observation is also consistent with what has been shown in previous studies [20,24,31]. Except the seed match type 1t8GU, all of the other types of seed matches in 3'UTRs have larger signal-to-noise ratios than their counterparts located in other gene regions.

**Table 1:** Signal-to-noise ratio, weight and proportion of different types of seed matches in different regions.

	2t8A1	2t8	2t7A1	2t7	1t8GU	
Number of matches (miRWalk)	1,235	3,105	2,657	7,296	3,734	Promoters
	171	628	463	1,594	730	5'UTRs
	1,153	3,116	2,729	6,494	3,343	CDSs
	2,366	4,103	3,204	6,920	3,797	3'UTRs

Number of matches (Average of 50 times random shuffle)	911	2,639	2,422	7,014	3,068	Promoters
	141	489	391	1,415	547	5'UTRs
	904	2,331	2,331	5,997	2,655	CDSs
	1,069	2,593	2,513	6,060	3,175	3'UTRs

Signal-to-noise ratio	1.355	1.177	1.097	1.040	1.217	Promoters
	1.213	1.284	1.184	1.126	1.333	5'UTRs
	1.275	1.337	1.171	1.083	1.259	CDSs
	2.214	1.583	1.275	1.142	1.196	3'UTRs

Weight	0.293	0.146	0.080	0.033	0.179	Promoters
	0.176	0.234	0.151	0.104	0.275	5'UTRs
	0.227	0.277	0.141	0.068	0.214	CDSs
	1.000	0.480	0.227	0.117	0.161	3'UTRs

Proportion (miRWalk)	7%	17%	15%	40%	21%	Promoters
	5%	18%	13%	44%	20%	5'UTRs
	7%	19%	16%	39%	20%	CDSs
	12%	20%	16%	34%	19%	3'UTRs

Proportion (Average of 50 times shuffle)	6%	16%	15%	44%	19%	Promoters
	5%	16%	13%	47%	18%	5'UTRs
	6%	16%	16%	42%	19%	CDSs
	7%	17%	16%	39%	21%	3'UTRs

The number of matches in miRWalk dataset is the real number of each seed match type in each target region. The number of matches from random shuffles is the average number of each type of seed match over 50 randomly shuffled mRNA sequences. The signal-to-noise ratio is the ratio of these two numbers. The weight is then calculated via the equation (1). The proportion is the percentage of a specific seed match type in each target region.

In CDSs, type 2t8 has the most significant signal-to-noise ratio, while the ratios for seed type 2t8 > 2t7A1 > 2t7, which is similar as those calculated for 3'UTRs. However, type 2t8 is more significant than 2t8A1 and 1t8GU is more significant than 2t7A1, deviated from what we have seen in 3'UTR. These results together demonstrate that the mechanism underlying miRNA target recognition and regulation in CDSs may be different from that in 3'UTRs.

In 5'UTRs, type 1t8GU has the most significant signal-to-noise ratio, while the order of other types of seed matches is similar as that in CDSs. This indicates that the GU wobble pair may play a much more important role in 5'UTRs relative to its effects in other gene regions.

For promoters, the order of the signal-to-noise ratios of four different canonical seed matches is similar to that in 3'UTRs, while 1t8GU type has the second highest ratio. Type 2t7 has the lowest ratio close to 1. These show that promoters are the least effective regions, but they cannot be ignored [32].

A recent study has shown that miRNA binding sites in CDSs mediate smaller regulation than 3'UTRs binding, and there may be possible interactions between targets sites in CDSs and 3'UTRs [33]. Another recent research study has also demonstrated that miRNA targets sites in CDSs can effectively inhibit translation while sites located in 3'UTRs are more efficient at triggering mRNAs degradation [34].

The proportion of five types of seed matches in each of the four gene regions are given in Table I. For the 50 random shuffled mRNA sequences, the distributions of the seed matches are similar among different regions, whereas, the proportion of seed match type 2t8A1 in 3'UTRs is much higher than that in other regions, based on the miRWalk data. Since type 2t8A1 is the most rigorous seed match type, it suggests that miRNA targets in 3'UTRs have more selection pressure.

### Comparison with other computational target identification methods

To verify the robustness of our method, we applied it on an independent benchmark dataset obtained by the pSILAC method [5] and evaluated how our target predictions correlate with the results in the pSILAC dataset. To achieve a comparable predicted number of targets with other well-known methods such as TargetScanS and PicTar, we set the cut-off values $\text{Δ}{{\text{G}}_{\text{duplex}}}_{{}_{\text{cutoff}}}=-\text{15}.0\text{ kcal}/\text{mol}$ and $\text{ΔΔ}{\text{G}}_{\text{cutoff}}=-\text{1}0.0\;\text{kcal}/\text{mol}$, respectively.

**Figure 3:**
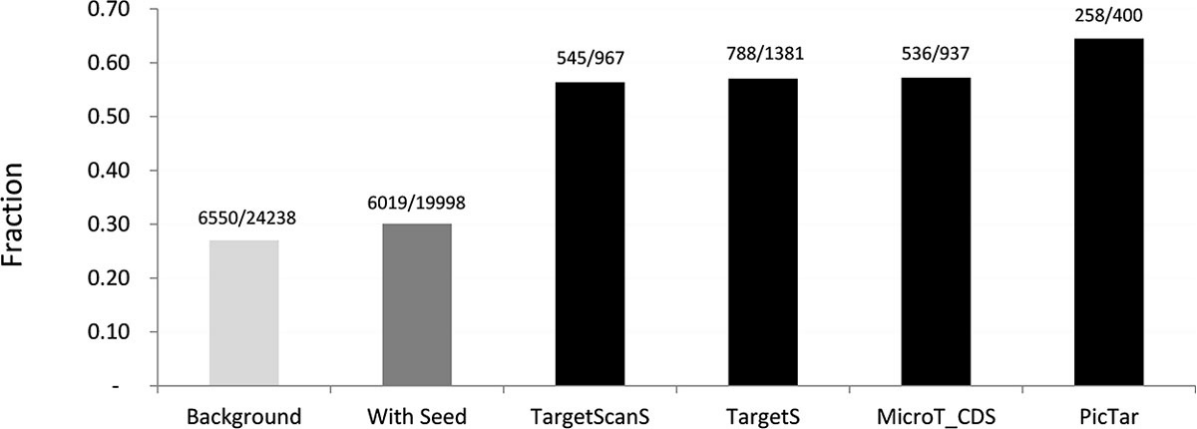
**Performance comparison of different miRNA target prediction methods**. The fraction of predicted targets with down regulated protein production in the pSILAC dataset.

Figure 3 shows the ratio of the fraction of predicted miRNA targets for which protein production was down regulated in the pSILAC dataset. We compared the performance of our method with that of most well-known target prediction tools including TargetScanS [8], PicTar [9] and MicroT_CDS [11]. While both TargetScanS and PicTar limit target site searching in 3'UTR of mRNAs, the MicroT_CDS includes CDSs in its target searching. From the results, we can see that, our method can predict ~47% more targets than the TargetScanS and MicroT_CDS, while achieving a comparable accuracy level of 57%. Although PicTar yielded the highest accuracy (64%) among all the methods we compared, it only identified about one third of the true targets that our method predicts. If we set more stringent cutoff values to $\text{Δ}{{\text{G}}_{duplex}}_{{}_{cutoff}}=-\text{25}.0\text{ kcal}/\text{mol}$ and $\text{ΔΔ}{\text{G}}_{cutoff}=-\text{14}.0\text{ kcal}/\text{mol}$, we achieve the same accuracy level of 64% (303/473) as PicTar with 17% more true positive targets identified, indicating the superiority of our method to PicTar. In the pSILAC dataset, when we just considered canonical seed matches in the 3'UTRs, we achieved an accuracy of 62% (430/692) based on the overlap of predicted target with the pSILAC data. While adding the 1t8GU non-canonical seed match, we identify 36 more true positive targets, with an accuracy of 61% (466/758). As we extended our target site searching region to include the CDSs, the predicted targets maintain an accuracy level of 61% (650/1071), while 184 more true positive targets were identified. Therefore, we can significantly increase the number of targets by including the CDSs, while maintaining high prediction accuracy. Continuing to extend the target searching region to 5'UTRs, we maintained the predicted accuracy at 61% (660/1088), with 10 more true positive targets being added. Finally, when we extended our target searching region to include the promoters, the fraction of overlap reduced to 57% (788/1381); however, it added 128 more true positive targets as indicated by pSILAC. These results show that, for miRNA targeting, CDSs and 5'UTRs might be similarly significant compared to 3'UTRs. The promoters might not be as effective as 3'UTRs, but it is important to include these regions to avoid missing a large number of true positive targets.

It has been shown previously that evolutionary conservation of target sites is a very important feature for improving the accuracy of target identification. To evaluate the effect of this feature in miRNA target prediction, we simply imported the conservation score of different seed match types calculated by phastCons [35] and set a cutoff value to identify the miRNA targets. The incorporation of target site conservation information indeed improved the accuracy of our method with an overlap of 65% (396/605) in pSILAC dataset, which is the highest accuracy among the state-of-the-art algorithms we investigated in our study. However it missed many true targets, no matter how we relaxed the stringency of the cutoff values for other features, namely $\text{Δ}{{\text{G}}_{\text{duplex}}}_{{}_{\text{cutoff}}}$ and $\text{ΔΔ}{\text{G}}_{\text{cutoff}}$. Therefore, we chose not to incorporate the evolutionary conservation information in our method to achieve high prediction coverage.

In the past decade, machine learning methods have been widely used to predict miRNA targets [15-18,21,36,37]. Besides the seed type matches and thermodynamic features, the most important and widely used features are structure features [16,37], such as single nucleotide composition, di-nucleotide composition, or frequency of base pair interaction. To compare the different features, we applied the Random Forests (RF) method [38] to evaluate the importance of each feature in miRNA target prediction. A set of 81 miRNA-targeting site context specific features was extracted. The features were mainly divided into seed and out-seed regions (the regions immediately connected to the seed matches). Each feature is the weighted sum of the seed matches located in the 3'UTRs, CDSs, 5'UTRs and promoters. Table 2 lists the importance index calculated by the Package 'RandomForest' in R (http://www.r-project.org) with the positives (down regulated proteins in response to miRNA overexpression) and negatives (other proteins measured) identified in the pSILAC dataset. From the table we can see that the two most important features are $\text{Δ}{\text{G}}_{\text{duplex}}$ and ΔΔG, which are consistent with the conclusion in previously published literature [19]. These two features are also the only two parameters used in our method TargetS for defining the true miRNA targets.

**Table 2:** Importance of different features.

Features	Importance
number of all kinds of seed matches	23.28
$\text{Δ}G_{duplex}$	79.11
ΔΔG	78.01
frequency of outseed A composition	67.96
frequency of outseed C composition	47.08
frequency of outseed G composition	49.72
frequency of outseed U composition	59.65
frequency of outseed AA composition	60.37
frequency of outseed AC composition	50.38
frequency of outseed AG composition	52.15
frequency of outseed AU composition	62.19
frequency of outseed CA composition	52.26
frequency of outseed CC composition	47.73
frequency of outseed CG composition	36.09
frequency of outseed CU composition	48.86
frequency of outseed GA composition	50.30
frequency of outseed GC composition	46.81
frequency of outseed GG composition	50.71
frequency of outseed GU composition	51.48
frequency of outseed UA composition	55.21
frequency of outseed UC composition	49.67
frequency of outseed UG composition	53.42
frequency of outseed UU composition	53.22
frequency of seed A composition	23.54
frequency of seed C composition	19.62
frequency of seed G composition	9.80
frequency of seed U composition	18.49
frequency of seed AA composition	3.72
frequency of seed AC composition	6.82
frequency of seed AG composition	3.41
frequency of seed AU composition	7.42
frequency of seed CA composition	10.57
frequency of seed CC composition	6.14
frequency of seed CG composition	0.35
frequency of seed CU composition	7.80
frequency of seed GA composition	1.01
frequency of seed GC composition	9.82
frequency of seed GG composition	0.92
frequency of seed GU composition	5.35
frequency of seed UA composition	12.01
frequency of seed UC composition	5.67
frequency of seed UG composition	9.15
frequency of seed UU composition	8.86
frequency of seed AU nucleotide base pairing	28.55
frequency of seed UA nucleotide base pairing	15.95
frequency of seed GC nucleotide base pairing	7.54
frequency of seed CG nucleotide base pairing	19.06
frequency of seed GU nucleotide base pairing	3.21
frequency of seed UG nucleotide base pairing	6.05
Frequency of seed AU-AU Bi-Di-nucleotide base pairing	3.75
Frequency of seed AU-UA Bi-Di-nucleotide base pairing	6.80
Frequency of seed AU-GC Bi-Di-nucleotide base pairing	3.59
Frequency of seed AU-CG Bi-Di-nucleotide base pairing	5.80
Frequency of seed AU-GU Bi-Di-nucleotide base pairing	-
Frequency of seed AU-UG Bi-Di-nucleotide base pairing	2.64
Frequency of seed UA-AU Bi-Di-nucleotide base pairing	10.61
Frequency of seed UA-UA Bi-Di-nucleotide base pairing	5.95
Frequency of seed UA-GC Bi-Di-nucleotide base pairing	-
Frequency of seed UA-CG Bi-Di-nucleotide base pairing	7.86
Frequency of seed UA-GU Bi-Di-nucleotide base pairing	0.33
Frequency of seed UA-UG Bi-Di-nucleotide base pairing	2.08
Frequency of seed GC-AU Bi-Di-nucleotide base pairing	-
Frequency of seed GC-UA Bi-Di-nucleotide base pairing	7.14
Frequency of seed GC-GC Bi-Di-nucleotide base pairing	-
Frequency of seed GC-CG Bi-Di-nucleotide base pairing	3.37
Frequency of seed GC-GU Bi-Di-nucleotide base pairing	-
Frequency of seed GC-UG Bi-Di-nucleotide base pairing	1.89
Frequency of seed CG-AU Bi-Di-nucleotide base pairing	12.21
Frequency of seed CG-UA Bi-Di-nucleotide base pairing	4.91
Frequency of seed CG-GC Bi-Di-nucleotide base pairing	7.55
Frequency of seed CG-CG Bi-Di-nucleotide base pairing	7.72
Frequency of seed CG-GU Bi-Di-nucleotide base pairing	2.55
Frequency of seed CG-UG Bi-Di-nucleotide base pairing	2.33
Frequency of seed GU-AU Bi-Di-nucleotide base pairing	1.02
Frequency of seed GU-UA Bi-Di-nucleotide base pairing	3.04
Frequency of seed GU-GC Bi-Di-nucleotide base pairing	0.95
Frequency of seed GU-CG Bi-Di-nucleotide base pairing	0.23
Frequency of seed UG-AU Bi-Di-nucleotide base pairing	2.33
Frequency of seed UG-UA Bi-Di-nucleotide base pairing	1.72
Frequency of seed UG-GC Bi-Di-nucleotide base pairing	-
Frequency of seed UG-CG Bi-Di-nucleotide base pairing	3.50

Number of all kinds of seed matches is the sum of all the 5 different seed match types in all different 4 regions. All other features are the properties of each single miRNA-mRNA seed match site. The importance is calculated by the Random Forests method based on the miRWalk dataset as positive training data and its relative random shuffle pairs as negative training data.

**Figure 4:**
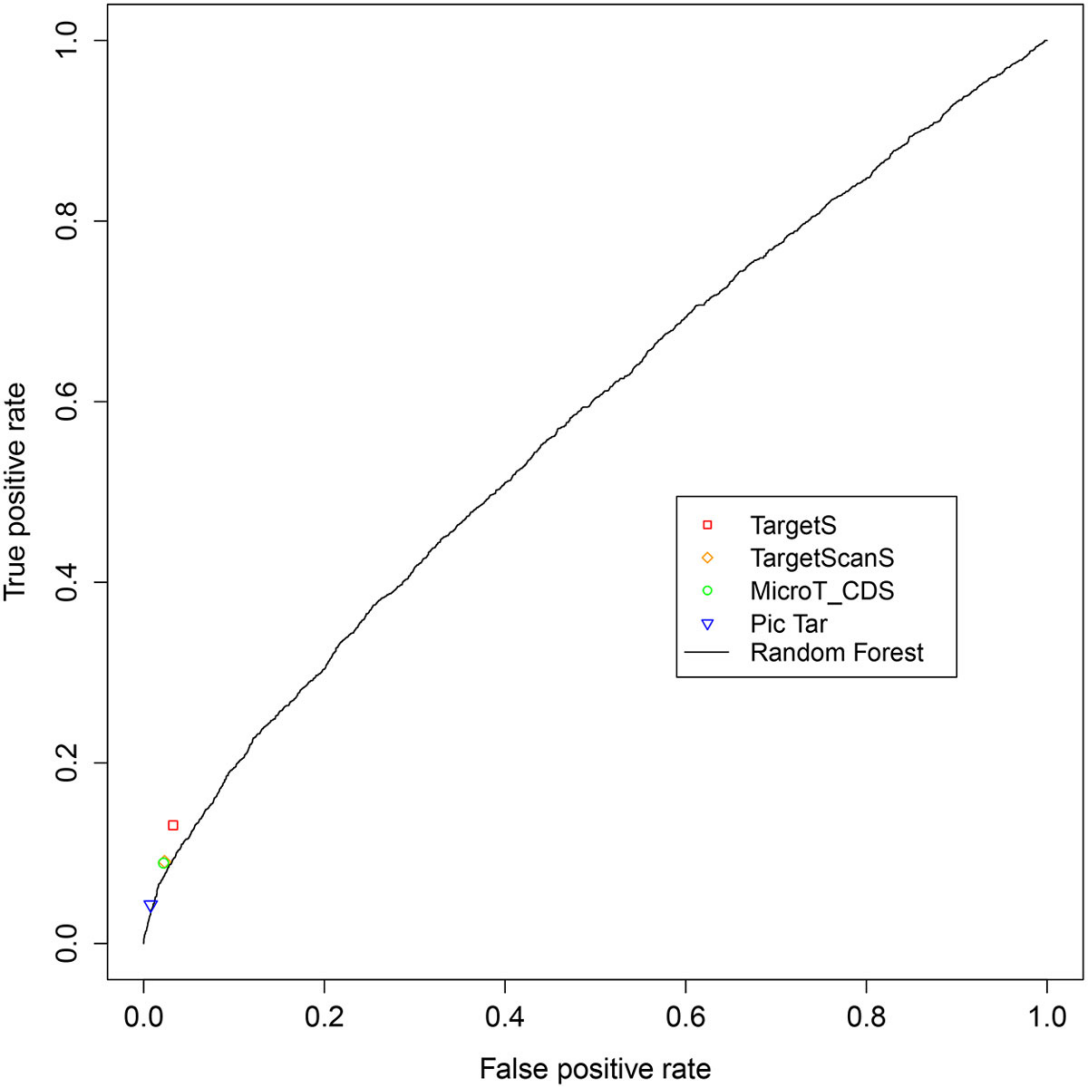
**ROC curve for Random Forest method**. The ROC curve for Random Forest obtained by 10-fold cross-validation on pSILAC dataset is shown with the results from other target prediction methods. The $\text{Δ}{\text{G}}_{duplex}$ and ΔΔG were used as input features for Random Forest.

We compared the performance of TargetS with that of the Random Forest (RF) method. To evaluate the results, we first applied a widely used k-fold cross-validation (CV) approach on the pSILAC data. The original sample is randomly partitioned into k equal size subsamples. Of the k subsamples, a single subsample is retained as the validation data for testing the model, and the remaining k−1 subsamples are used as training data. The cross-validation process is then repeated k times, with each of the k subsamples being used exactly once as the validation data. The k results from the folds can then be averaged to produce an overall estimation. The result of 10-fold cross validation on the pSILAC dataset by RF is shown in the ROC curve (Figure 4). With the top two features, $\text{Δ}{\text{G}}_{\text{duplex}}$ and ΔΔG as the input features, the performance of RF is comparable with PicTar, MicroT_CDS and TargetScanS, while our method TargetS is shown to significantly outperform all the methods we compared based on the assessment of sensitivity and specificity.

**Figure 5:**
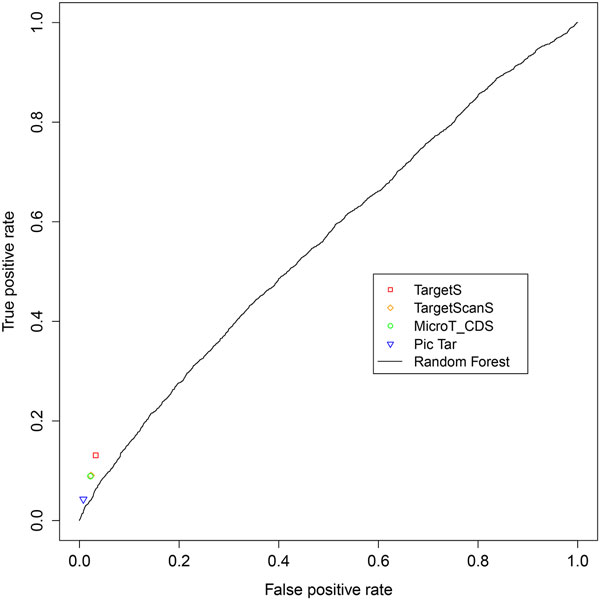
**ROC curve of independent testing**. The ROC curve for Random Forest with all the 81 features listed in Table 2. The model was trained on miRWalk dataset and tested on the independent pSILAC dataset.

Moreover, we note the performance of RF was evaluated based on the CV approach. The comparable performance of RF may be simply an artifact, due to the potential data overfitting effect caused by the CV. To better evaluate the performance of RF, we trained it using the experimentally verified miRNA-mRNA pairs in miRWalk as the positive training set, and the sequences generated by random shuffling as the negative training set. The trained model was then tested on the independent pSILAC dataset. The performance of RF decreased compared to other methods when an independent dataset was used for testing instead of performing CV on the same dataset (Figure 5). The disadvantage of machine learning methods lies on its requirement of a reliable negative training dataset, which is not currently available for most miRNAs. To overcome this problem, our TargetS method adopted a simple strategy to calculate the signal-to-noise ratio for seed matches using the experimentally verified miRWalk dataset. The ratios vary among different seed match types as well as their gene locations, and are used as the basis for assigning different weights for the parameters used in our method.

## Conclusions

In this paper, we have proposed a simple and novel computational method for miRNA target prediction (TargetS), which searches for miRNA target sites in either the 3'UTRs, CDSs, 5'UTRs or promoters. As mentioned, our method does not rely on evolutionary conservation, thus allowing the detection of species-specific interactions and making it suitable for analyzing un-conserved genomic sequences. We also include a non-canonical seed pairing type, namely the GU wobble pair as an alternative targeting criterion. The comparison results of TargetS with other methods were based on the independent pSILAC dataset, indicating that TargetS finds a significantly larger number of true miRNA targets at an accuracy level comparable with TargetScanS, PicTar and MicroT_CDS. We have developed a web-based tool that can easily access the human miRNA target prediction results from our TargetS method, with the miRNA name and/or gene name as the input. The user-friendly website is now available at: http://liubioinfolab.org/targetS/mirna.html. With the advent of large-scale sequencing and new crosslinking methods, more direct information of miRNAs and their targets' regulation will be obtained. Together with the information obtained from reliable computational prediction methods, the mechanism of miRNAs and their roles in regulating different important biological processes and molecular pathways can be further investigated. We hope such mechanistic insights will help us understand the progression of different types of diseases, and will lead to novel therapeutic strategies associated with miRNAs and their targets' regulation.

## Materials and methods

### Data

miRBase: The mature miRNAs sequences are downloaded from miRBase database [3]. There are more than 30,000 reported miRNAs entries, including 2,557 entries for human in the latest version (Release 20, 2013).

miRWalk: This dataset hosts experimentally verified miRNA-mRNA interactions as well as the information of genes, pathways, organs, diseases, cell lines, OMIM disorders and literature on miRNAs [39]. It includes 60,269 verified pairs of human miRNA-gene interactions that consist of 655 unique miRNAs and 3,028 unique genes.

pSILAC: A set of miRNA target genes identified by pSILAC (pulsed stable isotope labeling with amino acids in cell culture) method [5]. It measured changes in synthesis of several thousand proteins in response to miRNA transfection or endogenous miRNA knockdown for five miRNAs (hsa-miR-1, hsa-miR-16, hsa-miR-155, hsa-miR-30a and hsa-let-7b). This dataset has been widely used as a benchmark for evaluating computational miRNA target prediction programs and can be downloaded from http://psilac.mdc-berlin.de

### Sequence

The sequences of the promoters, 5'UTRs, CDSs and 3'UTRs for each gene in human have been downloaded from the UCSC Genomes database [40] using the UCSC Table Brower, version GRCh37/hg19. When there are multiple sequences available for a single gene (e.g. multiple UCSC IDs corresponding to a single gene name), the longest sequence was chosen for further analysis.

### Parameters considered in miRNA target prediction

Previously published methods [1,19,23] have shown that the most important features for miRNA target genes are 5′ seed matches of miRNA and thermodynamic stability of the miRNA-target duplex. We considered both features in our method when scoring each miRNA-mRNA pair.

For the first important feature, the types of canonical seed matches include 2t8A1 (requires Watson-Crick pairing to the 5' region of the miRNA on nucleotides 2 to 8 and the first nucleotide of target mRNAs being adenine), 2t8 (seed paring from position 2 to 8 in the 5' region of the miRNA), 2t7A1 (seed paring from position 2 to 7 with position 1 of target mRNA being adenine) and 2t7 (seed matches from position 2 to 7). However, many experimental results have shown that some 'non-seed' target sites such as single mismatches, GU wobbles, insertions or deletions in the seed-match regions are highly biologically functional as well [20,24,31]. Since insertions or deletions do not have a fixed format and it's hard to measure the significance of the signal, we just considered one non-canonical type of seed match, namely 1t8GU type (seed paring on positions 1 to 8 while allowing 1 GU wobble pair). So we have included five types of seed matches: 2t8A1, 2t8, 2t7A1, 2t7 and 1t8GU in our method (Figure 1).

The second important feature of targeting is thermodynamic stability. The binding energy between miRNA and the target mRNAs gained to form the miRNA-target duplex, $\text{Δ}{\text{G}}_{\text{duplex}}$ is an important base measurement of duplex stability. The lower the free energy gained from the formation of miRNA-target duplex, the stronger the binding structure is and the more likely it suggests a true target binding. Kertesz et al. (2007) also found that the accessibility energy, ΔΔG, which is the difference between the free energy, $\text{Δ}{\text{G}}_{\text{duplex}}$, and the free energy required to unpair the target-site nucleotides to make the target accessible to the miRNA, $\text{Δ}{\text{G}}_{\text{open}}$, has a strong correlation with the measured degree of miRNA-mediated translational repression [19]. So we took both the $\text{Δ}{\text{G}}_{\text{duplex}}$ and ΔΔG to measure thermodynamic stability of target binding in developing our method. The binding energy was calculated by RNAhybrid [41]. For each miRNA-mRNA pair, we calculated $\text{Δ}{\text{G}}_{\text{duplex}}$ using the miRNA sequence and 58 nucleotides flanking the seed match sites in the mRNA sequences, including the seed match sites, the 30 and 20 nucleotides immediately connected to the 5' and 3' of seed match, respectively, while $\text{Δ}{\text{G}}_{\text{open}}$ was calculated based on the 58 nucleotides in the mRNA sequence. We calculated the $\text{Δ}{\text{G}}_{\text{duplex}}$ and ΔΔG for all seed matches found in each miRNA-mRNA pair.

### Summarizing the free energy and the accessibility energy for each miRNA-mRNA pair

When summarizing the free energy $\left(\text{Δ}{\text{G}}_{\text{duplex}}\right)$and the accessibility energy (ΔΔG) for each miRNA-mRNA pair, we took into account all seed matches located in the entire mRNA sequence. Since different seed match types have been shown to correlate with different targeting efficacy (e.g. 2t8A1 > 2t8 > 2t7A1 > 2t7 in 3'UTR) [23], we proposed to assign different weights to each seed match according to their types and their location in the mRNA sequence. We first calculated the signal-to-noise ratio for each type of seed match located in different regions according to the miRWalk dataset. The miRNA sequences were extracted from miRBase, and the target mRNA sequences were downloaded from the UCSC Genome Browser. Based on the verified miRNA-mRNA pair in the miRWalk dataset, we counted the number of seed matches for each of the five different types in different gene regions, and then we randomly shuffled the mRNAs sequence 50 times and computed the average numbers of each type of these seed matches over 50 random shuffles. The seed match type 2t8A1 in 3'UTRs regions yielded the highest signal-to-noise ratio, so it was assigned a standard weight of 1. The weights of other seed match types were calculated as (1).

(1) $${\text{W}}_{\text{ij}}=\frac{\text{SN}{\text{R}}_{\text{ij}}-1}{\text{SN}{\text{R}}_{2\text{t}8\text{A}1\text{\_}3\text{'UTRs}}-1}$$

Where ${\text{W}}_{\text{ij}}$ indicates the weight of the seed match type i, located in the gene region j. $\text{SN}{\text{R}}_{\text{ij}}$ is the signal-to-noise ratio of type i seed match in gene region j and $\text{SN}{\text{R}}_{2\text{t}8\text{A}1\text{\_}3\text{'UTR}}$ is the signal-to-noise ratio of type 2t8A1 in 3'UTRs. Then we calculated the summarized $\text{Δ}{\text{G}}_{\text{duplex}}$ and ΔΔG for each miRNA-mRNA pair, as (2) and (3), respectively.

(2) $$\text{Total}\;\text{Δ}{\text{G}}_{\text{duplex}}=\overset{\text{n}}{\underset{\text{i}=1}{\sum }}\overset{\text{m}}{\underset{\text{j}=1}{\sum }}{\text{W}}_{\text{ij}}\text{*}\;\text{Δ}{\text{G}}_{\text{duplex}}$$

(3) $$\text{Total}\;\text{ΔΔ}\text{G}=\overset{\text{n}}{\underset{\text{i}=1}{\sum }}\overset{\text{m}}{\underset{\text{j}=1}{\sum }}{\text{W}}_{\text{ij}}\text{*}\;\text{ΔΔ}\text{G}$$

Where $\text{Δ}{\text{G}}_{\text{duplex}}$ is the binding energy of a miRNA-mRNA duplex. A weight of ${\text{W}}_{\text{ij}}$ is assigned if the pair contains the seed match of type i and the seed match is located in the gene region j. In our method, we considered five types of seed matches in each of the four gene regions (the promoter, 5'UTR, CDS, 3'UTR), so we have n = 5 and m = 4. Similarly, the accessibility energy (ΔΔG) gained for seed match of type i located in the region j is assigned the weight of ${\text{W}}_{\text{ij}}$ as well.

Then we set two cutoff values, $\text{Δ}{{\text{G}}_{\text{duplex}}}_{{}_{\text{cutoff}}}$ and $\text{ΔΔ}{\text{G}}_{\text{cutoff}}$. When the summarized $\text{Δ}{\text{G}}_{\text{duplex}}$ and ΔΔG are both less than their respective cutoff values, we label the mRNA as a putative target of the miRNA.

## Competing interests

The authors declare that they have no competing interests.

## Authors' contributions

WX and YL conceived of the idea and wrote the paper. WX performed the derivations, implemented the algorithm and prepared the data. FAS created the website and helped with writing the manuscript. ZW participated in the data analysis and results discussion. All authors read and approved the final manuscript.

## Declarations

The authors declare that all page charges for this article will be paid using the NIH grant R01 LM010022.

This article has been published as part of *BMC Bioinformatics *Volume 15 Supplement 7, 2014: Selected articles from the 10th Annual Biotechnology and Bioinformatics Symposium (BIOT 2013). The full contents of the supplement are available online at http://www.biomedcentral.com/bmcbioinformatics/supplements/15/S7
